# To Thrombolyse or Not to Thrombolyse: Two Years Experience of Thrombolysis of Sub-Massive Pulmonary Embolism in a District General Hospital

**DOI:** 10.7759/cureus.11359

**Published:** 2020-11-06

**Authors:** Muhammad Jawad, Caroline Apsey

**Affiliations:** 1 Acute Internal Medicine, Surrey and Sussex Healthcare National Health Service Trust, Redhill, GBR; 2 Internal Medicine, Lewisham and Greenwich National Health Service Trust, London, GBR

**Keywords:** systemic thrombolysis, submassive pulmonary embolism

## Abstract

Aims

Current British Thoracic Society (BTS) guidelines only recommend thrombolysis of pulmonary embolism (PE) in patients who are haemodynamically compromised. Newer evidence suggests a mortality benefit for the thrombolysis of sub-massive PE with right ventricular strain. We wanted to assess the outcome and safety of thrombolysis in patients with sub-massive PE in a DGH.

Methods

The notes for patients with sub-massive PE and thrombolysis from a two-year period were reviewed. Evidence of right ventricular strain and myocardial necrosis based on bedside echocardiography, computed tomography (CT) scan and troponin T were indications for thrombolysis.

Results

A total of 22 patients had thrombolysis of PE in the study period (56±14 years). Fourteen patients were classified as sub-massive PE (55±15 years). Out of eight patients who had thrombolysis of massive PE (58±14 years), three were initially classified as sub-massive PE but deteriorated within the next 48 hours and became haemodynamically unstable. In all patients, the diagnosis was confirmed with a CT pulmonary angiography (CTPA).

Mean troponin was 82 in the sub-massive PE group and 102 in the massive PE group. The clinical condition and haemodynamic of patients improved rapidly within a few hours after thrombolysis.

Post-thrombolysis echocardiography was performed, 17 patients had normal right ventricles with normal pulmonary arterial pressures.

Conclusion

Thrombolysis of sub-massive pulmonary embolism is feasible in a district general hospital and seems to be a safe procedure, particularly in younger patients. It results in rapid improvement in the clinical condition of patients with a small incidence of bleeding complications.

## Introduction

Pulmonary embolism (PE) is a common condition managed in district general hospitals with an annual incidence in the UK between 60-70/100,000 [1]. Morbidity and mortality rates vary widely. Pulmonary embolism is associated with increased mortality rates for up to three months after the index PE event [2].

Treatment varies depending on the severity of the presentation, with current British Thoracic Society (BTS) guidelines only recommending thrombolysis of PE in patients who are haemodynamically compromised (massive PE). In patients with normal blood pressure and no signs of shock on presentation, RV dysfunction provides indirect evidence of severe pulmonary arterial obstruction and impending haemodynamic failure [3]. Acute right ventricular pressure overload at diagnosis is an important determinant of the severity and early clinical outcome of pulmonary embolism [4].

A sub-massive PE, defined as the presence of right ventricular dysfunction and elevated troponin levels without evidence of hypotension [5], is typically managed with oral anticoagulation.

It is understood that thrombolysis results in faster improvements in pulmonary obstruction, pulmonary vascular resistance and pulmonary arterial pressure in patients with PE as compared with management with unfractionated heparin alone [5-6]. When imaged on echocardiography, these improvements are seen in conjunction with a reduction in right ventricle dilatation [5].

New evidence is suggesting that there may be a mortality benefit of thrombolysis for patients that have a sub-massive PE with right ventricular strain and myocardial damage. The Pulmonary Embolism Thrombolysis (PEITHO) trial [4] investigated the impact of thrombolytic therapy in patients with a sub-massive PE and found a significant reduction in the risk of patients suffering from haemodynamic collapse and reduction in overall mortality. However, the PEITHO trial did find an increased risk of severe side effects, including bleeding.

The European Society of Cardiology and European Respiratory Society (ESC/ERS) guidelines for the management of PE are still inconclusive on whether early thrombolysis for sub-massive PE has an impact on clinical symptoms and function upon long-term follow-up [5]. Therefore, further studies are required to investigate the role of thrombolysis in the management of sub-massive PE. Some clinicians advocate giving thrombolysis to patients with PE who are normotensive but with evidence of right heart strain or myocardial injury (e.g. on echocardiography, raised troponin) [7].

This article was previously presented as a poster: Jawad M, Dachsel M, Woosey D, Limb C, Wilde M: To thrombolyse or not to thrombolyse - Two years experience of thrombolysis of submassive pulmonary embolism in a District General Hospital. 8th International Society for Acute Medicine Conference. October 2, 2014.

## Materials and methods

The clinical notes for thrombolysed patients with sub-massive PE from a two-year period in one district general hospital were reviewed. All retrospective data collected were fully anonymised. Data were collected from patients who had been thrombolysed for submassive PE with indication documented of evidence of right ventricular strain based on bedside echocardiography and evidence of myocardial damage based on raised troponin T levels but not haemodynamically unstable. Notes were made of contraindications before thrombolysis and documentation of any complications that arose afterwards. None of the thrombolysed patients had contraindications to thrombolysis. Data analysis was performed using XLStat and Microsoft Excel (Microsoft Corporation, Redmond, WA). A Shapiro-Wilk test was used to check for Gaussian distribution. Gaussian distributed variables were shown as mean standard deviation and a student t-test was used to check for differences in the distributions. However, non-Gaussian distributed variables were shown as median (range) and a Mann-Whitney U test was used to test for differences in distribution.

## Results

**Figure 1:**
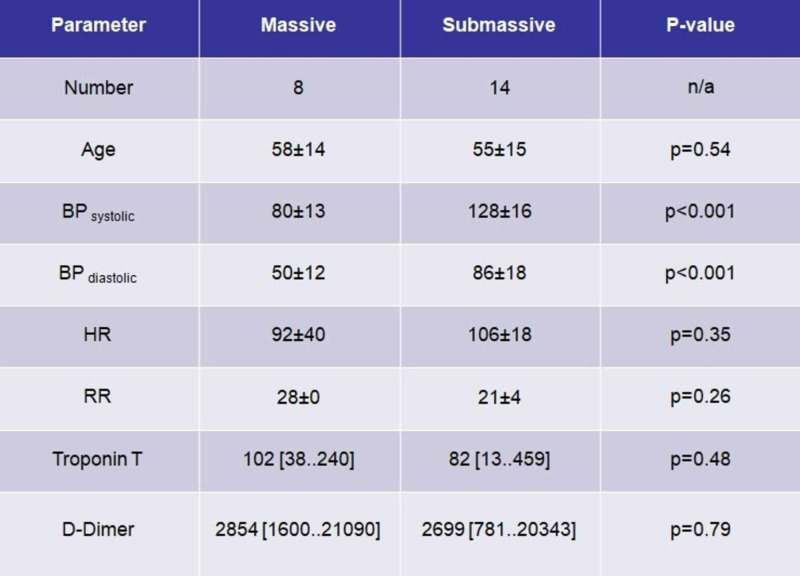
Demographics, observations and lab results

**Figure 2:**
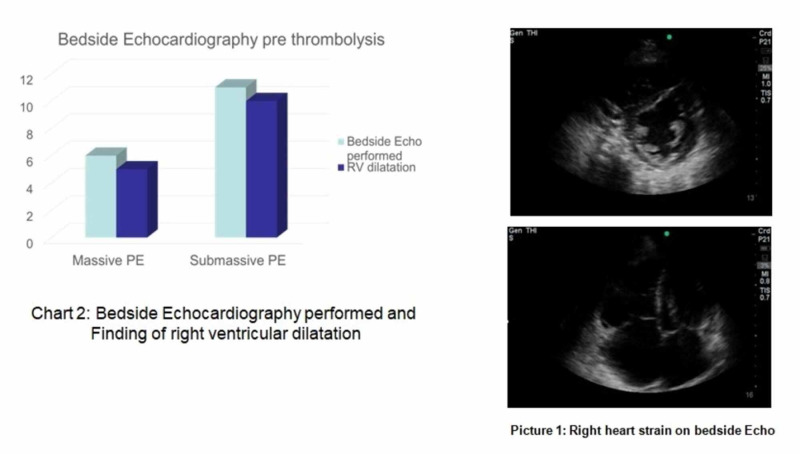
Bedside echocardiography and finding of right ventricular dilatation

**Figure 3:**
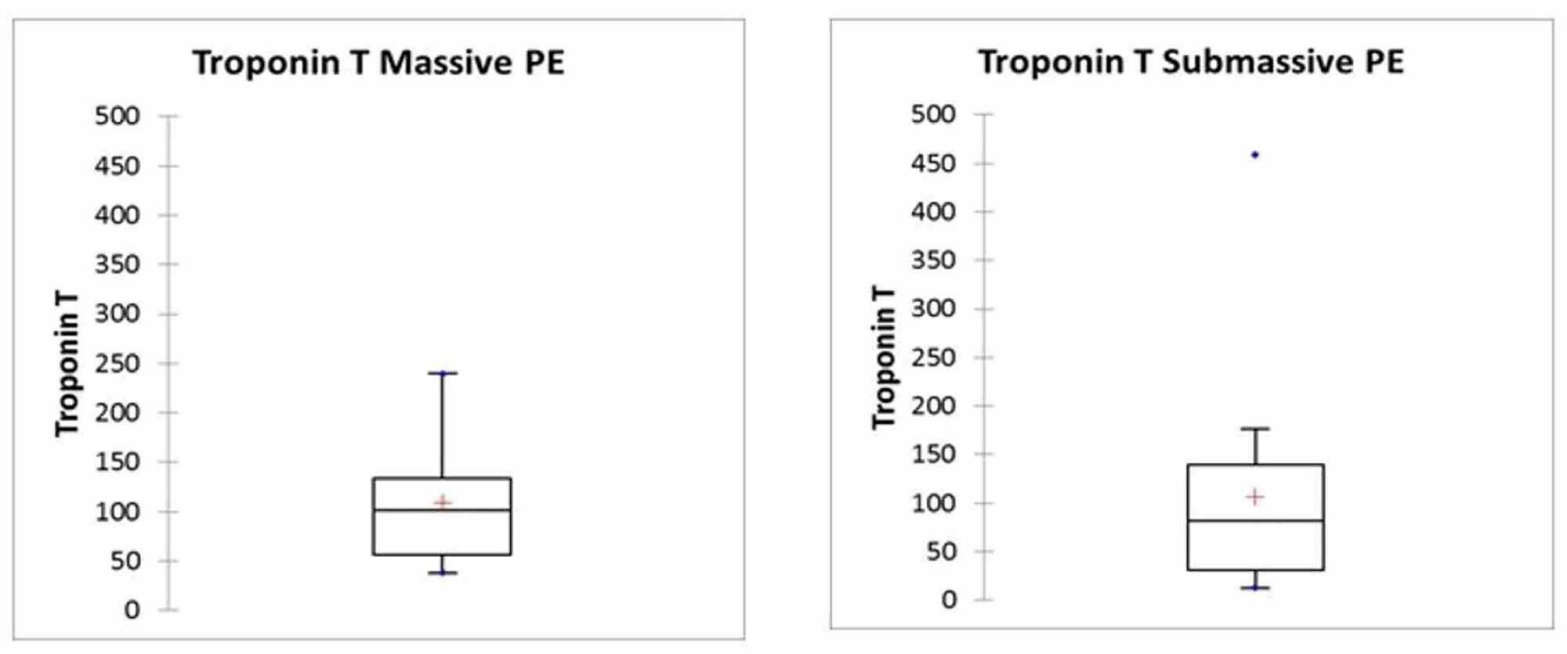
Box and whisker plot troponin T

A total number of 22 patients had thrombolysis for PE in the study period of two years (56±14 years). Seventeen patients were classified as sub-massive PE (54±14 years). Out of eight patients who had thrombolysis for massive PE (58±15years), three patients were initially classified as sub-massive PE but deteriorated within the next 48 hours and became haemodynamically unstable (Figure 1). In all patients, the diagnosis of PE was confirmed with a CTPA (Figure 2). Average troponin T was 102 ng/l (38 to 240 ng/l) in the massive PE group and 82 ng/l (13 to 459 ng/l) in the sub-massive PE group (p=0.48, see Figure 2, Chart 1). A reference range of troponin T of over 30 indicated myocardial damage (Figure 3). Pre-thrombolysis bedside echocardiograms were performed in six patients of the massive PE group. In five of the patients, a dilated right ventricle was documented. In the sub-massive group, 11 bedside echocardiograms were documented, with 10 showing an enlarged right ventricle (Figure 2, Chart 2). None of the bedside echocardiograms reported the easy detectable D-shaped septum sign in the parasternal short axis (PSAX) window (Figure 2, Picture 1).

Post-thrombolysis echocardiography was performed in 19 patients over the two-year period. Repeat echo was performed within a week of thrombolysis in massive PE and after six months in those thrombolysed for submassive PE. Seventeen patients had normal right ventricles with normal pulmonary arterial pressures.

There were no deaths recorded as a result of thrombolysis. Bleeding complications were found in three patients. Two of those were thrombolysed for submassive PE and both were above 75 years of age, and one patient was thrombolysed for massive PE within 24 hours of knee surgery and was not yet started on prophylactic heparin. One patient had gross haematuria (age 77 years), which settled quickly without the need for blood transfusion, one patient had bleeding from the knee surgical wound site requiring blood transfusion (58 years), and one patient had a 2.5 cm intracerebral haematoma, which resolved spontaneously (82 years). There was no history of previous stroke or head trauma in any of those patients. No bleeding complications resulted in mortality in the patient population studied.

## Discussion

Current BTS guidelines recommend only thrombolysis of massive PE [1]. However, Grifoni et al. show that 30% of patients with sub-massive PE showed signs of right ventricular dysfunction and out of these patients, 10% progressed to shock with a 5% mortality rate [3]. The patients in our study with sub-massive PE had similar troponin T levels as the patients with massive PE. Ninety per cent of performed bedside echocardiograms showed signs of right heart dilatation.

Three patients were originally classified as sub-massive PE but deteriorated to haemodynamic instability and then underwent thrombolysis for massive PE. There is a possibility that more patients with sub-massive PE could have become haemodynamically compromised if thrombolysis wasn’t given early. In fact, patients with elevated cardiac markers and right heart strain on echocardiogram or CTPA might be able to compensate for some time before deterioration into shock. It is then probably unsurprising that Chatterjee et al., in a recent meta-analysis, found a mortality benefit for the thrombolysis of sub-massive PE (OR of death 0.48; 95% CI, 0.25-0.92) [2].

Chatterjee S et al. found that, particularly in patients less than 65 years of age, thrombolysis is safe [2]. In our study, out of all the patients who had thrombolysis there were only three bleeding events. In particular, looking at patients less than 75 years of age, as done in the PEITHO trial [4], there was only one patient postoperatively after a total knee replacement that had a haemorrhage from the operation site within 24 hours of surgery. The other two patients were over 75 years of age and had no contraindications for thrombolysis This demonstrates that age is an important relative contraindication to be considered for the use of thrombolysis to manage sub-massive PE. Additionally, on follow-up echocardiography, 90% of patients had no evidence of right heart strain.

## Conclusions

Thrombolysis of submassive PE is feasible in a district general hospital and has proved to be a safe procedure, particularly in younger patients less than 75 years of age. It might result in treating patients with a high potential of haemodynamic decompensation early and might decrease death and morbidity. This study recommends that more research should be conducted into the safety and acceptability of thrombolysing submassive PEs at both district general and teaching hospitals across the UK.
